# Validation of a Deep Learning–Based Model to Predict Lung Cancer Risk Using Chest Radiographs and Electronic Medical Record Data

**DOI:** 10.1001/jamanetworkopen.2022.48793

**Published:** 2022-12-28

**Authors:** Vineet K. Raghu, Anika S. Walia, Aniket N. Zinzuwadia, Reece J. Goiffon, Jo-Anne O. Shepard, Hugo J. W. L. Aerts, Inga T. Lennes, Michael T. Lu

**Affiliations:** 1 Cardiovascular Imaging Research Center, Department of Radiology, Massachusetts General Hospital & Harvard Medical School, Boston, Massachusetts; 2 Program for Artificial Intelligence in Medicine, Brigham and Women’s Hospital & Harvard Medical School, Boston, Massachusetts; 3 Division of Abdominal Imaging, Department of Radiology, Massachusetts General Hospital & Harvard Medical School, Boston, Massachusetts; 4 Division of Thoracic Imaging and Intervention, Department of Radiology, Massachusetts General Hospital & Harvard Medical School, Boston, Massachusetts; 5 Department of Radiology and Nuclear Medicine, CARIM School for Cardiovascular Diseases and GROW School for Oncology and Reproduction, Maastricht University, the Netherlands; 6 Division of Hematology and Oncology, Department of Medicine, Massachusetts General Hospital & Harvard Medical School, Boston, Massachusetts

## Abstract

**Importance:**

Lung cancer screening with chest computed tomography (CT) prevents lung cancer death; however, fewer than 5% of eligible Americans are screened. CXR-LC, an open-source deep learning tool that estimates lung cancer risk from existing chest radiograph images and commonly available electronic medical record (EMR) data, may enable automated identification of high-risk patients as a step toward improving lung cancer screening participation.

**Objective:**

To validate CXR-LC using EMR data to identify individuals at high-risk for lung cancer to complement 2022 US Centers for Medicare & Medicaid Services (CMS) lung cancer screening eligibility guidelines.

**Design, Setting, and Participants:**

This prognostic study compared CXR-LC estimates with CMS screening guidelines using patient data from a large US hospital system. Included participants were persons who currently or formerly smoked cigarettes with an outpatient posterior-anterior chest radiograph between January 1, 2013, and December 31, 2014, with no history of lung cancer or screening CT. Data analysis was performed between May 2021 and June 2022.

**Exposures:**

CXR-LC lung cancer screening eligibility (previously defined as having a 3.297% or greater 12-year risk) based on inputs (chest radiograph image, age, sex, and whether currently smoking) extracted from the EMR.

**Main Outcomes and Measures:**

6-year incident lung cancer.

**Results:**

A total of 14 737 persons were included in the study population (mean [SD] age, 62.6 [6.8] years; 7154 [48.5%] male; 204 [1.4%] Asian, 1051 [7.3%] Black, 432 [2.9%] Hispanic, 12 330 [85.2%] White) with a 2.4% rate of incident lung cancer over 6 years (361 patients with cancer). CMS eligibility could be determined in 6277 patients (42.6%) using smoking pack-year and quit-date from the EMR. Patients eligible by both CXR-LC and 2022 CMS criteria had a high rate of lung cancer (83 of 974 patients [8.5%]), higher than those eligible by 2022 CMS criteria alone (5 of 177 patients [2.8%]; *P* < .001). Patients eligible by CXR-LC but not 2022 CMS criteria also had a high 6-year incidence of lung cancer (121 of 3703 [3.3%]). In the 8460 cases (57.4%) where CMS eligibility was unknown, CXR-LC eligible patients had a 5-fold higher rate of lung cancer than ineligible (127 of 5177 [2.5%] vs 18 of 2283 [0.5%]; *P* < .001). Similar results were found in subgroups, including female patients and Black persons.

**Conclusions and Relevance:**

Using routine chest radiographs and other data automatically extracted from the EMR, CXR-LC identified high-risk individuals who may benefit from lung cancer screening CT.

## Key Points

**Question:**

Can a deep learning model that uses chest radiographs and other electronic medical record data identify high-risk individuals for lung cancer screening, to complement the Centers for Medicare & Medicaid Services lung cancer screening computed tomography eligibility criteria?

**Findings:**

In this prognostic study of electronic medical record data of 14 737 patients in the Mass General Brigham health system, a deep learning model identified persons at high risk for lung cancer including those missed by Medicare lung cancer screening eligibility criteria.

**Meaning:**

These results suggest that a deep learning model can help identify high-risk individuals who may benefit from lung cancer screening with chest computed tomography.

## Teaser

This prognostic study of patients in a large US health system validates a risk prediction tool to identify persons at high risk for lung cancer who may not be included in Medicare screening eligibility criteria.

## Introduction

Lung cancer is the leading cause of cancer death globally^1^; in the US it results in more deaths than the next 3 cancer types combined.^2^ Screening for lung cancer with chest computed tomography (CT) reduces lung cancer mortality by 20% to 24%.^3,4^ In 2015, US Centers for Medicare & Medicaid Services (CMS) established coverage for lung cancer screening for individuals aged 55 to 77 years with a smoking history of 30 pack-years or more (packs of cigarettes smoked per day × years of smoking) who are currently smoking cigarettes or quit within the past 15 years.^5^ However, 2015 CMS guidelines have significant limitations.^6,7^ More than 50% of lung cancers occur in those ineligible by 2015 CMS criteria, and less than 6% of the 8 million eligible Americans are screened,^8,9^ a dismal participation rate compared with breast (64%) and colorectal (63%) cancer.^10^ The 2015 CMS criteria may perpetuate disparities in lung cancer screening^11,12,13^ by excluding female patients and patients from minoritized racial groups that are at high risk despite younger age^14^ and lesser cigarette smoking exposure.^15^

The 2021 US Preventive Services Task Force (USPSTF) recommendation expanded eligibility to include those as young as 50 years and to lower the pack-year threshold from 30 or more to 20. This is estimated to expand eligibility to 15 million Americans.^9,16,17^ In 2022, CMS adopted these criteria, in part to reduce disparities in screening eligibility.^18^ However, the new 2022 guidelines do not address the critical issue with lung cancer screening—the poor participation compared with other cancer screening tests. There are many reasons why doctors and patients do not participate in lung cancer screening, including difficulty remembering eligibility criteria, lack of time to obtain a smoking history, and absence of electronic medical record (EMR) reminders.^7^ Automated EMR alerts identifying high-risk patients likely to benefit from screening are a promising way to improve screening participation^7^; however, smoking pack-years and years since quitting are often not documented in the EMR, or are documented inaccurately.^19,20^ This has resulted in underreporting of eligibility by 54%.^21^ An automated system that identifies high-risk individuals using universally available EMR data would enable automated alerting of primary care professionals as a step toward improving lung cancer screening participation.

To this end, we released a free, open source, deep learning–based risk prediction model, called CXR-LC,^22^ that identifies high-risk individuals who smoke cigarettes for lung cancer screening CT based on readily available EMR data: age, sex, current cigarette smoking status, and a chest radiograph image. In 2 cancer screening trials, CXR-LC missed 30.7% fewer lung cancers than the 2015 CMS criteria while screening the same number of people. Limitations included a majority (86.7%) non-Hispanic White population and inclusion of asymptomatic lung cancer screening trial populations having lung cancer screening chest radiographs, rather than the far more common radiographs obtained through routine clinical care. CXR-LC was externally validated in a South Korean health check-up cohort; however, this sample was 95% male, had only 1 year of follow-up, and used annual check-up chest radiographs, which are uncommon in the US.^23^

Our vision is to implement CXR-LC as an automated EMR tool to flag high-risk persons who smoke and improve lung cancer screening CT participation. The aim of this study was to retrospectively evaluate whether CXR-LC can use data commonly available in the EMR (age, sex, whether currently smoking, existing chest radiograph images obtained through routine clinical care) to identify individuals at high risk of lung cancer. The intent is to improve current low rates of lung cancer screening CT participation. This is a retrospective, external validation of the CXR-LC risk prediction model in outpatients from a single hospital system, with 6-year incident lung cancer as the primary outcome. In secondary analyses, we tested the performance of CXR-LC in subgroups with historical lung cancer screening disparities, including persons of self-reported Black racial identity and female individuals.

## Methods

### Study Cohort

**Figure 1.**
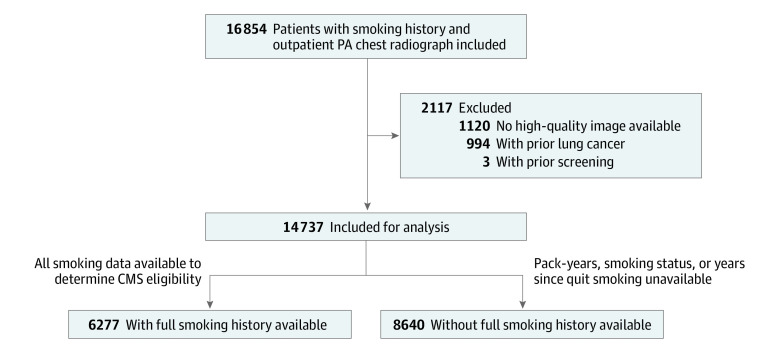
CONSORT Diagram CMS indicates US Centers for Medicare & Medicaid Services; PA, posterior-anterior.

Our cohort consisted of patients aged 50 to 80 years who had a history of cigarette smoking documented in the electronic medical record (EMR) and an outpatient posterior-anterior chest radiograph taken at a Mass General Brigham hospital in 2013 or 2014 (Figure 1). This period was chosen to capture the era immediately prior to the 2015 CMS lung cancer screening decision and to allow time for a 6-year follow-up period for incident lung cancer. Exclusion criteria included a past lung cancer diagnosis, previous lung cancer screening CT, or unavailable chest radiograph image through the Picture Archiving and Communication System (PACS). We performed analyses in (1) a cohort where smoking data was available to determine CMS eligibility (6277 individuals) and (2) a cohort where pack-years, smoking status, or years since quit smoking was missing and CMS eligibility could not be determined (8460 individuals). This study was approved by the Mass General Brigham institutional review board with a waiver of informed consent for retrospective analysis of deidentified data. The study followed Transparent Reporting of a Multivariable Prediction Model for Individual Prognosis or Diagnosis (TRIPOD) reporting guideline reporting guidelines for a risk prediction model validation study.

### CXR-LC and Chest Radiographs

CXR-LC^22^ is a free, open-source convolutional neural network that takes a radiograph image and characteristics on age, sex, and whether the person is currently smoking as inputs. CXR-LC outputs a probability score (between 0 and 1) of 12-year lung cancer risk. The risk prediction model was developed in over 64 000 individuals from the Prostate, Lung, Colorectal, and Ovarian (PLCO) cancer screening trial^24,25^ and validated in over 10 000 additional individuals from PLCO and the National Lung Screening Trial (NLST).^3^ Radiographs in PLCO and NLST were obtained from asymptomatic volunteers; here we tested CXR-LC on a patient cohort where radiographs were obtained for clinical indications through routine care.

This study is an external validation of CXR-LC. The existing published version of CXR-LC was used without any modification.^22,26^ CXR-LC risk thresholds defined in the original publication were applied. CXR-LC was originally developed in the PLCO cancer screening trial, which enrolled in 1993 to 2001 from 10 US sites, none of which were part of the Mass General Brigham hospital system or in the New England region.^27^ Existing frontal upright posterior-anterior chest radiograph images were obtained from the PACS system. When a patient had multiple radiographs during the study period, the earliest image was used. Chest radiograph preprocessing steps are described in eMethods in the Supplement.

### Smoking History, Risk Factors, Race, and Ethnicity

Smoking history and other risk factors were determined from the EMR. A previously described longitudinal algorithm^28^ extracted smoking status (current or former), pack-years, and years since quitting smoking at the time the image was taken for each patient. If smoking status could not be determined, patients were assumed to not be currently smoking. History of chronic obstructive pulmonary disease (COPD) was defined using *International Classification of Diseases, Ninth Revision *(*ICD-9*) and *International Statistical Classification of Diseases and Related Health Problems, Tenth Revision *(*ICD-10*) codes.^29,30^ Race and ethnicity were self-reported and defined according to the National Institutes of Health Policy on Reporting Race and Ethnicity Data.^31^

### Outcomes

The primary outcome was incident lung cancer in the 6 years after chest radiograph based on *ICD-9* and *ICD-10* codes for primary lung cancers obtained from the EMR. To reduce errors, diagnoses were included only if an *ICD* code appeared more than once.^32^ All identified lung cancer events were then confirmed via manual review of discharge summaries, clinic notes, radiology reports, and pathology reports. All-cause mortality was determined using the Social Security Master Death Index and the Mass General Brigham death registration system.

### CMS Eligibility Criteria

We compared 2015 CMS lung cancer screening eligibility criteria against 2022 CMS criteria; 2015 CMS criteria was defined as adults aged 55 to 77 years with at least a 30 pack-year smoking history (packs per day × years of smoking) who were either currently smoking or were 15 years or less since quitting. In 2022, CMS expanded eligibility to those ages 50 to 77 years with at least a 20 pack-year smoking history who were currently smoking or within 15 years of quitting.^5^ As the 2022 CMS criteria are the new standard of care,^7^ we focused our evaluation of CXR-LC against the 2022 criteria.

### CXR-LC Risk Score and Threshold for Screening Eligibility

Because this is an external validation of CXR-LC, we used the same risk categories and thresholds described in the original CXR-LC publication.^22^ Continuous CXR-LC probability scores from 0% to 100% were converted to an ordinal scale based on 12-year incident lung cancer probability thresholds from PLCO (low, ≤2%; indeterminate, ≥2% to 3.297%; high, ≥3.297% to 8%; and very-high, ≥8%).^22^ Individuals were considered eligible for screening if the CXR-LC 12-year risk probability was 3.297% or higher. This threshold was chosen in the original report as it yielded an equally sized screening population as the 2015 CMS eligibility criteria in the PLCO trial.

### Statistical Analysis

#### Test Characteristics of CXR-LC Eligibility

Among the subgroup of patients where CMS eligibility could be determined, sensitivity and specificity of CXR-LC eligibility were compared with the 2015 and 2022 CMS criteria using a McNemar test.^33^ Venn diagrams and 2-by-2 tables present 6-year incident lung cancer rates based on the 2015 CMS, 2022 CMS, and CXR-LC eligibility criteria. We calculated 95% confidence intervals for sensitivity, specificity, and positive predictive value (PPV) using the epiR package version 2.0.52.^34^ The threshold for statistical significance was *P* < .05 in 2-sided tests.

#### Decision Curves and Subgroup Analysis

Decision curves are a tool to estimate the net benefit of prediction models across possible risk thresholds.^35,36^ This metric calculates the benefits of true positives weighed against the harms of false positives based on the chosen risk threshold. For example, choosing a 2% threshold for lung cancer screening suggests that a clinician is willing to screen 100 individuals to catch 2 lung cancers. Thus, at this threshold, missing an instance of lung cancer by not recommending screening (ie, a false negative) is 49 times worse than screening a patient who will not develop lung cancer (ie, a false positive). The advantage of this analysis is that it weighs harms and benefits across a spectrum of screening strategies and risk thresholds.^37^

We present decision curves for CXR-LC, 2022 CMS, and 2015 CMS eligibility criteria, and null scenarios of screening everyone and screening no one. We applied decision curve analysis to subgroups stratified by sex, history of COPD, smoking status, and White vs Black race.

## Results

### Demographic Characteristics, Smoking History, Lung Cancer, and Lung Cancer Screening

A total of 14 737 patients were included with a mean (SD) age of 62.6 (6.8) years. Within the cohort, 7154 patients (48.5%) were male, 204 (1.4%) Asian, 1051 (7.3%) Black, 432 (2.9%) Hispanic, and 12 330 (85.2%) White (Table 1). Using smoking data extracted from the EMR, 2022 CMS eligibility criteria could be determined for 6277 patients (42.6%), and of these patients 1151 (18.3%) were eligible for lung cancer screening by 2022 CMS criteria (714 of 7755 [9.2%] for the 2015 CMS criteria). The 6-year incident lung cancer rate for the overall cohort was 2.4% (361 of 14 737). Among 2015 CMS eligible patients, only 84 of 714 patients (11.8%) underwent lung cancer screening CT in the 6 years after their chest radiograph. Those with smoking information to determine CMS eligibility were demographically similar to those whose CMS eligibility could not be determined; however, they had a 2-fold increase in 6-year lung cancers (216 of 6277 [3.4%] vs 145 of 8460 [1.7%]) and a higher rate of prevalent COPD and emphysema (1157 of 6277 [18.4%] vs 882 of 8460 [10.4%]).

**Table 1.** Cohort Demographics, Smoking History, and Outcomes for Patients With or Without Smoking Information to Determine CMS Eligibility

Characteristics	Patients, No./total No. (%)		
	Full cohort (N = 14 737)	CMS eligibility available (N = 6277)	CMS eligibility unavailable (N = 8460)
Age, mean (SD), y	62.6 (6.8)	62.4 (6.8)	62.8 (6.7)
Sex			
Female	7583/14 737 (51.5)	3324/6277 (53.0)	4259/8460 (50.3)
Male	7154/14 737 (48.5)	2953/6277 (47.0)	4201/8460 (49.7)
Race			
Asian	204/14 473 (1.4)	71/5973 (1.2)	135/8156 (1.7)
Black	1051/14 473 (7.3)	522/5973 (8.7)	531/8156 (6.5)
White	12 330/14 473 (85.2)	5149/5973 (86.2)	7196/8156 (88.2)
Other^a^	456/14 473 (3.2)	241/5973 (4.0)	285/8156 (3.5)
Hispanic ethnicity	432/14 737 (2.9)	220/6277 (3.5)	212/8460 (2.5)
Current smoking	3433/12 807 (26.8)	2255/6277 (35.9)	914/8460 (10.8)
Years since smoking cessation, mean (SD)	19.5 (13.2)	19.7 (14.1)	6.15 (8.1)
Pack-years, mean (SD)	18.6 (23.5)	26.3 (28.4)	33.1 (26.4)
2022 CMS lung cancer screening-eligible	1151/6277 (18.3)	1151/6277 (18.3)	NA
2015 CMS-eligible	714/7755 (9.2)	714/6277 (11.4)	NA
6-y Lung cancer screening rate	501/14 737 (3.4)	301/6277 (4.8)	200/8460 (2.4)
Lung cancer screening rate among 2015-CMS eligible	84/714 (11.8)	84/714 (11.8)	NA
6-y Lung cancer incidence	361/14 737 (2.4)	216/6277 (3.4)	145/8460 (1.7)
6-y All-cause mortality	1284/14 272 (9.0)	703/6045 (11.6)	581/8227 (7.1)
Lung nodule present on radiograph	1662/14 737 (11.3)	653/6277 (10.4)	1009/8460 (11.9)
Presence of COPD and emphysema	2039/14 737(13.8)	1157/6277 (18.4)	882/8460 (10.4)

Abbreviation: CMS, US Centers for Medicare & Medicaid Services; COPD, chronic obstructive pulmonary disease; NA, not applicable.

^a^ Including American Indian and Alaskan Native, Native Hawaiian and Pacific Islander, and other racial and ethnic groups not specifically documented.

When compared with the clinical trial data used to develop CXR-LC, we found that our study cohort had a similar rate of 6-year lung cancer incidence (361 of 14 737 [2.4%] vs 418 of 22 711 [1.8%]) (eTable 1 in the Supplement). However, this cohort had more female individuals (7583 of 14 737 [51.5%] vs 9051 of 22 711 [39.9%]), lower pack-years (mean [SD] pack-years, 18.6 [23.5] vs 35.2 [29.0]) and CMS eligibility (1151 of 6277 [18.3%] vs 9852 of 22 694 [43.4%]), and higher rates of COPD and emphysema (2039 of 14 737 [13.8%] vs 869 of 22 640 [3.8%]).

### CXR-LC Eligibility at Predefined Screening Thresholds

Patients were considered CXR-LC eligible if they had a 12-year risk greater than 3.297%; this threshold was previously chosen so that the CXR-LC eligible population was the same size as the 2015 CMS eligible population in the PLCO trial. CXR-LC identified 974 of 1151 patients (84.6%) of the 2022 CMS population as high-risk, and caught 83 of 88 six-year lung cancers (94.3%) among CMS eligible patients (Table 2). Patients that were ineligible by CMS, but eligible by CXR-LC had a high rate of 6-year incident lung cancer (121 of 3703 [3.3%]). Among the 8460 patients where CMS eligibility could not be determined based on EMR data, CXR-LC eligible patients had a 5-fold higher rate of lung cancer than those that were ineligible (6-year lung cancer rate: CXR-LC eligible, 127 of 5177 [2.5%] vs CXR-LC ineligible, 18 of 3283 [0.5%]; *P* < .001). Six-year lung cancer rates by CXR-LC and CMS eligibility were similar to those observed in prior clinical trial cohorts used to validate the CXR-LC model; however, incidence in CXR-LC and 2022 CMS eligible populations were higher in this study (83 of 974 [8.5%] vs 43 of 1376 [3.1%]) (eTable 4 in the Supplement). Patients eligible by both criteria were at higher risk than those eligible by CMS alone in secondary analyses excluding patients with lung nodules or cancers diagnosed within 9 months (eFigure 5 in the Supplement) and in Black patients (eFigure 6 in the Supplement). Cumulative incidence curves by CXR-LC and CMS eligibility show accumulation of risk across all 6 years of follow-up for CXR-LC and CMS eligible groups (eFigure 7 in the Supplement).

**Table 2.** Six-Year Lung Cancer Rates by CXR-LC and 2022 CMS Eligibility

Category	Patients, No./total No. (%)			
	CMS eligible	CMS ineligible	CMS eligibility unknown	All
CXR-LC				
Eligible	83/974 (8.5)	121/3703 (3.3)	127/5177 (2.5)	331/9854 (3.3)
Ineligible	5/177 (2.8)	7/1423 (0.5)	18/3283 (0.5)	30/4883 (0.6)
All	88/1151 (7.6)	128/5126 (2.5)	145/8460 (1.7)	361/14 737 (2.4)

Abbreviation: CMS, Centers for Medicare & Medicaid Services.

In the population where CMS eligibility could be determined from the EMR, CXR-LC caught 204 cancers for 216 patients (94.4%) of 6-year lung cancers while screening 4677 of 6277 patients (74.5%) (Table 3). For those where CMS eligibility could not be determined, CXR-LC caught 127 of 145 lung cancers (87.6%) while screening 5177 of 8640 patients (61.2%). Similar results were found in Black patients (eTable 5 in the Supplement).

**Table 3.** Test Statistics for CXR-LC, 2015 CMS, and 2022 CMS Eligibility Criteria for 6-Year Incident Lung Cancer

Characteristics	% (95% CI)				
	2015 CMS		2022 CMS	CXR-LC	
**Smoking history available (n = 6277)**					
Sensitivity	31.0 (24.9-37.6)	40.7 (34.1-47.6)			94.4 (90.5-97.1)
Specificity	89.3 (88.5-90.1)	82.5 (81.5-83.4)			26.2 (25.1-27.3)
PPV	9.4 (7.3-11.8)	7.6 (6.2-9.3)			4.4 (3.8-5.0)
NPV	97.3 (96.9-97.7)	97.5 (97.0-97.9)			99.3 (98.7-99.6)
Total eligible for screening, No./total No. (%)	714/6277 (11.4)	1151/6277 (18.3)			4677/6277 (74.5)
Lung cancers included, No./total No. (%)	67/216 (31.0)	88/216 (40.7)			204/216 (94.4)
**Smoking history unavailable (n = 8460)**					
Sensitivity	NA	NA			87.6 (81.1-92.5)
Specificity	NA	NA			39.3 (38.2-40.3)
PPV	NA	NA			2.5 (2.0-2.9)
NPV	NA	NA			99.5 (99.1-99.7)
Total eligible for screening, No./total No. (%)	NA	NA			5177/8640 (61.2)
Lung cancers included, No./total No. (%)	NA	NA			127/145 (87.6)

Abbreviations: CMS, Centers for Medicare & Medicaid Services; NA, not applicable; NPV, negative predictive value; PPV, positive predictive value.

### Decision Curve Analysis

**Figure 2.**
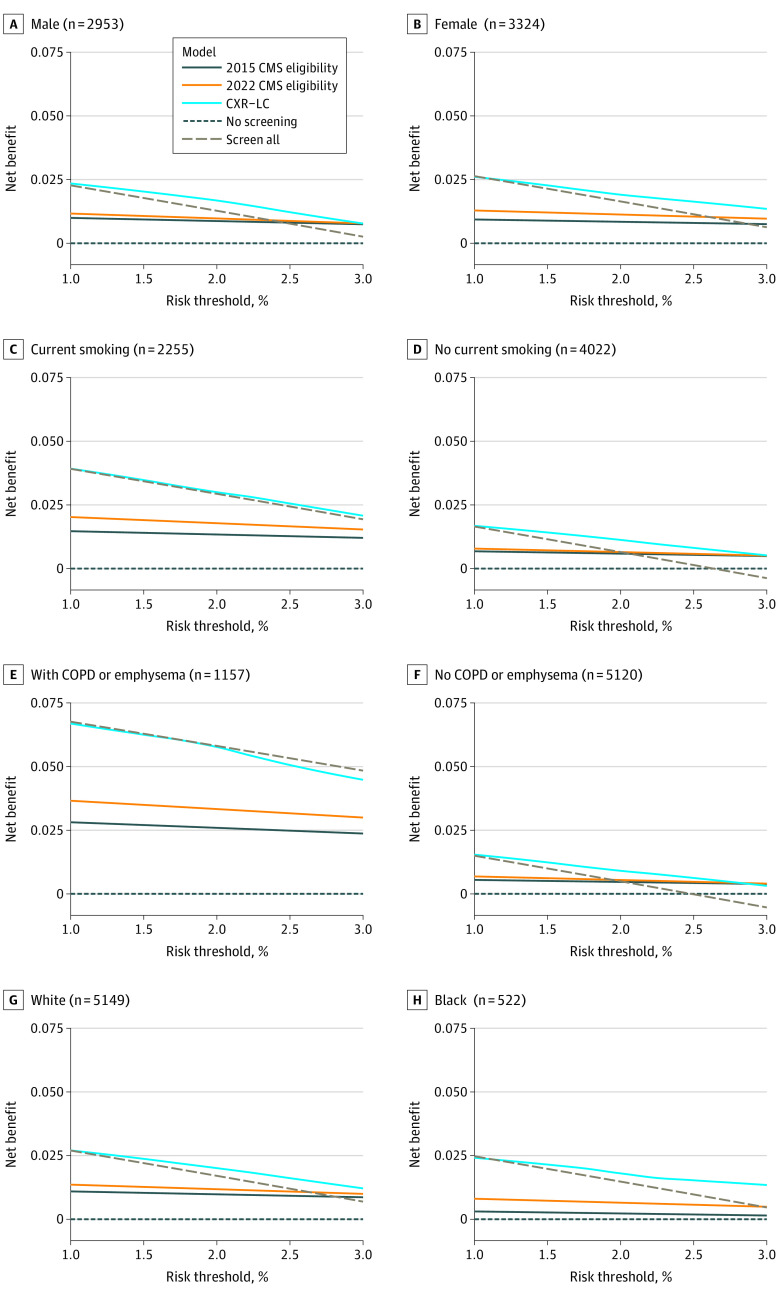
Decision Curves for Lung Cancer Screening Based on an Outcome of 6-Year Incident Lung Cancer in Subgroups Defined by Sex, History of Chronic Obstructive Pulmonary Disease (COPD), Smoking Status, and Self-reported Race CMS indicates US Centers for Medicare & Medicaid Services.

Decision curves for 6-year lung cancer are provided in Figure 2. National Comprehensive Cancer Network (NCCN) practice guidelines suggest lung cancer screening CT for individuals with a 1.3% or higher 6-year risk of lung cancer.^38^ Risk-based screening thresholds of greater than 1.5%^39^ and greater than 2%^6^ have also been proposed. Across these risk thresholds (1.3% to 2.0% for 6-year lung cancer), CXR-LC had equal or higher net benefit than 2022 and 2015 CMS eligibility criteria, and baseline strategies of screening everyone and screening no one. This was robust in subgroups defined by sex, smoking status, history of COPD, and self-reported race. In most subgroups, the 2022 CMS eligibility criteria had higher net benefit than 2015 CMS criteria regardless of the chosen risk threshold. Additional results including a comparison between 2015 and 2022 CMS criteria (eFigure 1) and discrimination and calibration of CXR-LC (eTables 2 and 3 and eFigures 2 through 4) are available in the Supplement.

## Discussion

In this study, we externally validated a deep learning model (CXR-LC) to identify persons at high risk for 6-year lung cancer who may benefit from lung cancer screening CT. Our major findings were (1) only 43% of patients had the EMR pack-year and quit-year data necessary to determine CMS eligibility, (2) CXR-LC identified 85% of 2022 CMS eligible patients and 94% of incident lung cancers, (3) CXR-LC eligible patients had a high incidence of lung cancer above the NCCN 1.3% or higher 6-year risk threshold, including in patients ineligible by 2022 CMS criteria (3.3%) and when CMS eligibility could not be determined (2.5%), and (4) CXR-LC was superior to 2022 CMS criteria on decision curve analysis, including in female and Black persons.

We designed CXR-LC as a pragmatic tool to automatically flag high-risk persons using inputs (chest radiograph image, age, sex, whether currently smoking) readily available in the EMR. The underlying concept is that there is information on the pixels of the chest radiograph image about lung cancer risk that can substitute for in-depth smoking information. Most modern health systems store radiograph images in a PACS attached to the EMR.^40^ Chest radiography is one of the most common medical tests; age and sex are embedded in the metadata header of radiograph images. In the US, the CMS Meaningful Use regulation mandates documentation of smoking status and multiple studies have shown that smoking status is available in over 80% of patients.^20,41,42^ When smoking history is unavailable, CXR-LC predictions could be provided using both former and current smoking status as input to allow the choice of the correct prediction. Others have developed lung cancer risk prediction models using numerous EMR inputs such as *ICD* codes, medications, spirometry, and other factors.^43,44^ To facilitate comparison with these models, we released CXR-LC as free open-source software.^26^ We envision CXR-LC as an automated system that runs in the background of the EMR. High-risk individuals would trigger an alert to the primary care provider to perform a targeted interview to determine smoking history and discuss lung cancer screening. For the 25% of US residents that do not have a primary care physician,^45^ CXR-LC alerts could provide an additional point during care to prompt a discussion of lung cancer screening for those receiving only episodic care in urgent, emergency, or subspecialty settings.

The CMS eligibility criteria have been reported to miss most lung cancers; in our cohort 2022 CMS criteria captured only 41% (88 of 216) of 6-year lung cancers. In the 47% of patients without pack-year or quit date EMR data to determine CMS eligibility, there were 145 additional incident lung cancers. In contrast, CXR-LC identified 91% (248 of 273) of lung cancers that would have been missed by 2022 CMS criteria. This raises an important question for CXR-LC—what should be done for patients who are CXR-LC eligible but not 2022 CMS eligible? In our data these patients had a 3.3% 6-year lung cancer rate, above the 1.3% to 2% risk thresholds reported as appropriate for screening CT. The practical issue is that Medicare and most insurance companies base payment decisions on CMS criteria, and may not pay for lung cancer screening CT in CMS-ineligible patients. This issue persists for any risk score that does not match CMS guidelines, and will not be resolved by this study. In our cohort, only 11% of persons who we could confirm were CMS-eligible completed a lung cancer screening CT in the 6-year follow-up period, in line with screening rates reported for Massachusetts (12%).^46^ Given this low rate, any steps to improve lung cancer screening participation would be valuable, even if only in the CMS-eligible population. CXR-LC identified 85% (974 of 1151) of the 2022 CMS eligible patients. Patients eligible by both criteria had a high (8.5%) 6-year lung cancer incidence—lung cancer screening CT should be prioritized in these patients.

Six-year incident lung cancer was very common (2.4%) in our cohort. The 2013 NCCN guidelines recommend lung cancer screening CT in persons with a 6-year lung cancer risk higher than 1.3%^38^; other authors have proposed thresholds of 1.5%^39^ and 2%^6^ for screening. Regardless of CMS eligibility, those that were CXR-LC eligible had 6-year incident lung cancer rates of 2.5% or higher, well above these thresholds. However, due to this high lung cancer rate, CXR-LC classified 67% of the population as screening eligible while capturing 91.7% of lung cancers. While the positive CXR-LC rate is justified by the 3.4% observed lung cancer rate, we acknowledge that alert fatigue is a potential issue.^47^ For this reason, we provide a set of results using a higher risk threshold (ie, “very high risk” in the CXR-LC model) (eFigures 8-10 in the Supplement). This higher threshold reduced the CXR-LC eligible population to 31.5% of the cohort, but still captured 63.9% of lung cancers in those where CMS eligibility could be determined and 62.8% in those without smoking information.

### Limitations

Limitations of this study should be considered. This was a retrospective study of patients who had a chest radiograph taken at a Mass General Brigham site in the Boston area; performance may differ when tested prospectively or in other locations. Our cohort was 85% non-Hispanic White; future studies need to validate CXR-LC in diverse populations. CXR-LC requires a chest radiograph image as input; patients without an image available were not included in this study. Based on prior estimates, 40% to 59% of adults who smoke cigarettes had a chest radiograph in the past 3 years.^22^ Lung cancer outcomes were based on *ICD* coding and were manually confirmed using pathology reports, clinic notes, and discharge summaries. This process may not capture cancers outside of our hospital system. We chose to use radiographs taken in 2013 to 2014 to ensure 6-year follow-up; however, this was prior to the 2015 CMS approval for lung cancer screening and the 2022 CMS expansion of eligibility, and may not reflect the current lung cancer screening landscape. Smoking history extracted from the EMR is often inaccurate,^28^ which could detract from the observed performance of both CXR-LC and CMS eligibility criteria. In practice, we anticipate that clinicians will use the CXR-LC alert as a prompt to discuss lung cancer screening with high-risk patients and obtain an accurate smoking history. CXR-LC was developed and validated in persons who currently or formerly smoked. Risk prediction in never-smokers is also needed and should be the focus of future investigation. Deep learning convolutional neural networks have been described as a “black box,”^48^ in that it is difficult to explain how a prediction is made for an individual patient. To address this, we used association analyses to show that the output of CXR-LC is primarily associated with reasonable correlates of lung cancer risk: age, COPD prevalence, and smoking history (eFigure 11 in the Supplement). A criticism of lung cancer risk prediction models is that they identify older and frailer individuals as high risk, who may be least likely to benefit from treatment after a diagnosis of lung cancer.^49^ This problem of overdiagnosis would need to be addressed in a clinical trial.

## Conclusions

This prognostic study externally validated CXR-LC, an open-source deep learning tool that identifies persons at high 6-year risk of incident lung cancer, using existing routine CXRs and basic data (age, sex, whether currently smoking) automatically extracted from a clinical EMR. Future work is necessary to determine whether implementation of CXR-LC–based alerts into the EMR would improve lung cancer screening CT participation, to complement CMS lung cancer screening eligibility criteria.

## Notes

Supplement.
eMethods.

eResults.

eReferences.
**eTable 1.** Demographics, Smoking History, Lung Cancer Screening and Outcomes of Patients in the Current Study Cohort, and in the Original Model Development Cohort **eFigure 1.** Lung Cancer Rates by 2015 CMS and 2022 CMS Eligibility Criteria **eTable 2.** Discrimination for 6-year Incident Lung Cancer **eTable 3.** Discrimination for 6-year Incident Lung Cancer in Subgroups by Sex and Self-reported Race **eFigure 2.** Absolute Calibration Plots of CXR-LC Estimated Risk vs Observed Lung Cancer Rate **eFigure 3.** Relative Calibration Plots of CXR-LC Estimated Risk vs. Percentage Calibration Error of Observed Lung Cancers **eFigure 4.** Association Between Ordinal CXR-LC Risk Groups and 6-year Lung Cancer Risk **eTable 4.** Six-year Lung Cancer Rates by CXR-LC and 2022 CMS Eligibility Criteria Across Model Development and Validation Cohorts **eFigure 5.** Lung Cancer Rates by CXR-LC and 2022 CMS Eligibility Criteria, Removing Positive Radiograph Screens and Those With Confirmed Lung Cancer Within 9 Months **eFigure 6.** Lung Cancer Rates by CXR-LC and 2022 CMS Eligibility Criteria in Black Patients **eFigure 7.** Cumulative Lung Cancer Incidence by CXR-LC and CMS Eligibility Criteria **eTable 5.** Test Statistics for CXR-LC and 2015 and 2022 CMS Eligibility Criteria for 6-year Incident Lung Cancer in Black Patients With Smoking History **eTable 6.** Test Statistics for 6-year Lung Cancer Rate by 2015 and 2022 CMS Eligibility and CXR-LC Using a Risk Threshold to Match the Specificity of 2022 CMS Criteria **eFigure 8.** Lung Cancer Rates by CXR-LC Very-High and 2022 CMS Eligibility Criteria **eFigure 9.** Lung Cancer Rates by CXR-LC Very-High and 2022 CMS Eligibility Criteria Removing Positive Radiograph Screens and Those With Confirmed Lung Cancer Within 9 Months **eFigure 10.** Lung Cancer Rates by CXR-LC Very-High and 2022 CMS Eligibility Criteria in Black Patients **eFigure 11.** Spearman Correlation Between the CXR-LC Probability Output and Prevalent Risk Factors and Pack-year Smoking History vs. CXR-LC Risk Probability
