# Supplementary material for: Validation of a Deep Learning–Based Model to Predict Lung Cancer Risk Using Chest Radiographs and Electronic Medical Record Data
Source: JAMA Netw Open. 2022 Dec 28;5(12):e2248793. doi: 10.1001/jamanetworkopen.2022.48793 (PMC9857639; doi:10.1001/jamanetworkopen.2022.48793)
Supplement: Supplement. — eMethods. eResults. eReferences. eTable 1. Demographics, Smoking History, Lung Cancer Screening and Outcomes of Patients in the Current Study Cohort, and in the Original Model Development Cohort eFigure 1. Lung Cancer Rates by 2015 CMS and 2022 CMS Eligibility Criteria eTable 2. Discrimination for 6-year Incident Lung Cancer eTable 3. Discrimination for 6-year Incident Lung Cancer in Subgroups by Sex and Self-reported Race eFigure 2. Absolute Calibration Plots of CXR-LC Estimated Risk vs Observed Lung Cancer Rate eFigure 3. Relative Calibration Plots of CXR-LC Estimated Risk vs. Percentage Calibration Error of Observed Lung Cancers eFigure 4. Association Between Ordinal CXR-LC Risk Groups and 6-year Lung Cancer Risk eTable 4. Six-year Lung Cancer Rates by CXR-LC and 2022 CMS Eligibility Criteria Across Model Development and Validation Cohorts eFigure 5. Lung Cancer Rates by CXR-LC and 2022 CMS Eligibility Criteria, Removing Positive Radiograph Screens and Those With Confirmed Lung Cancer Within 9 Months eFigure 6. Lung Cancer Rates by CXR-LC and 2022 CMS Eligibility Criteria in Black Patients eFigure 7. Cumulative Lung Cancer Incidence by CXR-LC and CMS Eligibility Criteria eTable 5. Test Statistics for CXR-LC and 2015 and 2022 CMS Eligibility Criteria for 6-year Incident Lung Cancer in Black Patients With Smoking History eTable 6. Test Statistics for 6-year Lung Cancer Rate by 2015 and 2022 CMS Eligibility and CXR-LC Using a Risk Threshold to Match the Specificity of 2022 CMS Criteria eFigure 8. Lung Cancer Rates by CXR-LC Very-High and 2022 CMS Eligibility Criteria eFigure 9. Lung Cancer Rates by CXR-LC Very-High and 2022 CMS Eligibility Criteria Removing Positive Radiograph Screens and Those With Confirmed Lung Cancer Within 9 Months eFigure 10. Lung Cancer Rates by CXR-LC Very-High and 2022 CMS Eligibility Criteria in Black Patients eFigure 11. Spearman Correlation Between the CXR-LC Probability Output and Prevalent Risk Factors and Pack-year Smoking History vs. [file jamanetwopen-e2248793-s001.pdf]

Supplemental Online Content

Raghu VK, Walia AS, Zinzuwadia AN, et al. Validation of a deep learning–based model to predict lung cancer risk using chest radiographs and electronic medical record data. *JAMA Netw Open.* 2022;5(12):e2248793. doi:10.1001/jamanetworkopen.2022.48793

eMethods.

eResults.

eReferences.

eTable 1. Demographics, Smoking History, Lung Cancer Screening and Outcomes of Patients in the Current Study Cohort, and in the Original Model Development Cohort

eFigure 1. Lung Cancer Rates by 2015 CMS and 2022 CMS Eligibility Criteria

eTable 2. Discrimination for 6-year Incident Lung Cancer

eTable 3. Discrimination for 6-year Incident Lung Cancer in Subgroups by Sex and Self-reported Race

eFigure 2. Absolute Calibration Plots of CXR-LC Estimated Risk vs Observed Lung Cancer Rate

eFigure 3. Relative Calibration Plots of CXR-LC Estimated Risk vs. Percentage Calibration Error of Observed Lung Cancers

eFigure 4. Association Between Ordinal CXR-LC Risk Groups and 6-year Lung Cancer Risk

eTable 4. Six-year Lung Cancer Rates by CXR-LC and 2022 CMS Eligibility Criteria Across Model Development and Validation Cohorts

eFigure 5. Lung Cancer Rates by CXR-LC and 2022 CMS Eligibility Criteria, Removing Positive Radiograph Screens and Those With Confirmed Lung Cancer Within 9 Months

eFigure 6. Lung Cancer Rates by CXR-LC and 2022 CMS Eligibility Criteria in Black Patients

eFigure 7. Cumulative Lung Cancer Incidence by CXR-LC and CMS Eligibility Criteria

eTable 5. Test Statistics for CXR-LC and 2015 and 2022 CMS Eligibility Criteria for 6-year Incident Lung Cancer in Black Patients With Smoking History

© 2022 Raghu VK et al. *JAMA Network Open.*

eTable 6. Test Statistics for 6-year Lung Cancer Rate by 2015 and 2022 CMS Eligibility and CXR-LC Using a Risk Threshold to Match the Specificity of 2022 CMS Criteria

eFigure 8. Lung Cancer Rates by CXR-LC Very-High and 2022 CMS Eligibility Criteria

eFigure 9. Lung Cancer Rates by CXR-LC Very-High and 2022 CMS Eligibility Criteria Removing Positive Radiograph Screens and Those With Confirmed Lung Cancer Within 9 Months

eFigure 10. Lung Cancer Rates by CXR-LC Very-High and 2022 CMS Eligibility Criteria in Black Patients

eFigure 11. Spearman Correlation Between the CXR-LC Probability Output and Prevalent Risk Factors and Pack-year Smoking History vs. CXR-LC Risk Probability

This supplemental material has been provided by the authors to give readers additional information about their work.

© 2022 Raghu VK et al. *JAMA Network Open*.

eMethods.

Study Cohort

All information was retrieved from our institution's Research Patient Data Registry (RPDR),[1] a centralized data repository developed to store clinical information from Mass General Brigham (MGB) hospitals including Massachusetts General Hospital, Brigham and Women's Hospital, North Shore Medical Center, Newton-Wellesley Hospital, Brigham and Women's Faulkner Hospital, Wentworth-Douglass Hospital, and >35 primary care clinics. This retrospective, observational study was approved by the institutional review board, with a waiver of informed consent. Data analysis was performed between May 2021 and June 2022.

Statistical Analysis (Cont.)

Discrimination and Calibration

Model discrimination for 6-year incident lung cancer was assessed using area under the receiver operating characteristic curve (AUC). DeLong's method was used to test for significant differences between the ROC curves for CXR-LC and the CMS criteria in patients where smoking data was available to determine CMS eligibility.[2]

© 2022 Raghu VK et al. *JAMA Network Open*.

Model calibration was assessed using calibration graphs and the Integrated Calibration Index (ICI),[3] a measure of the difference between observed and predicted rates of lung cancer. As CXR-LC predicts 12-year probabilities and the primary outcome in the current study was 6-year lung cancer, we divide CXR-LC probabilities by 2 as a simple approximation. Confidence intervals for the ICI were computed using 1,000 bootstrap samples. Discrimination and calibration of CXR-LC are reported separately for those where CMS eligibility could and could not be determined based on the available EMR data, and in subgroups of self-reported race and sex.

Survival Analysis

We assessed the association between the ordinal CXR-LC risk categories with time to incident lung cancer using Cox proportional hazards regression. Hazard ratios were adjusted for 2022 CMS eligibility, history of COPD, sex, and race.[4] Kaplan-Meier survival curves present lung cancer incidence by CXR-LC risk strata, accounting for deaths as a censoring event.

Chest Radiograph Images and Preprocessing

Images were downloaded in Digital Imaging Communications in Medicine (DICOM) format and converted to .png using the same image pre-processing steps used to develop CXR-LC (https://github.com/circ-ml/CXR-LC).[5] Poor quality CXR images were detected using a deep learning model trained to estimate view position from the radiograph image (https://github.com/circ-ml/CXR-View). Images were excluded if the model had less than 80% confidence that the image was a posterior-anterior CXR.

© 2022 Raghu VK et al. *JAMA Network Open*.

The text of the radiologists' reports of the CXRs was extracted from the EMR. Presence of lung nodules and other radiographic findings were extracted using the CheXpert-labeler software, an open-source tool that identifies radiographic findings from free-text reports.[6]

© 2022 Raghu VK et al. *JAMA Network Open*.

eResults.

2015 vs. 2022 CMS criteria

A Venn diagram comparing the 2015 vs. the expanded 2022 CMS screening eligibility criteria is given in Supplemental Figure 1. Those that were eligible by 2015 criteria had an 9.4% rate of lung cancer (67 cancers / 714 persons). The 2022 CMS criteria include an additional 437 patients with a high (4.8%) rate of lung cancer; however, those that are ineligible for screening by both criteria still have a 2.5% rate of lung cancer (128 cancers / 5,126 patients). Screening eligibility could not be determined in 6,982 persons due to missing smoking information in the EMR (47.4% of the cohort). These individuals had a 2.1% rate (147 cancers / 6,982 persons) of 6-year lung cancer.

Discrimination and Calibration of CXR-LC

Discrimination for incident lung cancer using CXR-LC risk probabilities vs. 2015 and 2022 CMS eligibility criteria are given in Supplemental Table 2. In 6,277 patients where smoking history was available, CXR-LC had higher discrimination for six-year lung cancer than 2022 CMS (Areas under the curve (AUC) 0.69 [0.66,0.73] vs. 0.62 [0.58,0.65], $p < 0.001$) and 2015 CMS (0.69 [0.66,0.73] vs. 0.60 [0.57,0.63], $p < 0.001$) criteria. In the 8,460 patients where CMS eligibility could not be determined from the EMR due to unavailable smoking history, CXR-LC retained high discrimination (0.74 [0.70,0.78]). Similar results were found after excluding patients with lung nodules and cancers occurring nine or fewer months after the CXR (i.e., people diagnosed with a lung nodule or lung cancer around the time of the CXR) and in subgroups defined by sex and black race (Supplemental Table 3).

© 2022 Raghu VK et al. *JAMA Network Open*.

As for calibration, CXR-LC slightly overestimated risk (Supplemental Figures 2,3) with an Integrated Calibration Index of 0.01 [0.008,0.012]. This trend was stronger in men (0.013 [0.010,0.017]), than in women (0.006 [0.003,0.010]). Additionally, the model overestimated risk in black patients (0.012 [0.008,0.016]). In particular, black patients with an estimated risk between 1-2% had an observed 6-year lung cancer rate of 0.5%.

Association of CXR-LC ordinal risk categories with incident lung cancer

Continuous CXR-LC risk scores were stratified into Low, Indeterminate, High, and Very-High risk groups based on previously derived thresholds from the PLCO lung cancer screening trial (see Methods).[5] CXR-LC risk groups were associated with incident lung cancer risk, and incident lung cancers continued to accumulate in the High and Very-High risk groups throughout the follow-up period (Supplemental Figure 4). After adjustment for race, sex, history of COPD, and 2022 CMS criteria, CXR-LC High and Very-High risk groups had a higher risk of incident lung cancer than the low-risk group (CXR-LC High aHR 3.14 [1.5,6.6], $p = 0.002$ and CXR-LC Very-High aHR 4.87 [2.4,10.1], $p < 0.001$).

© 2022 Raghu VK et al. *JAMA Network Open*.

CXR-LC Eligibility at predefined screening thresholds (Cont.)

To assess the performance of a lower sensitivity CXR-LC threshold for screening, we repeated these analyses in two ways. First, we determined a new threshold such that CXR-LC eligibility had equal specificity as 2022 CMS criteria and found that both criteria had similar specificity (Supplemental Table 6). Second, we treated only the Very-High risk group (>8% 6-year risk) as eligible for screening and all other risk groups as ineligible (Supplemental Figures 8-10). In patients with a documented smoking history, this strategy caught 63.9% of lung cancers (138 cancers / 216 patients) while screening 29.7% (1867 / 6277) of patients. In patients where CMS eligibility was unavailable, this strategy caught 62.8% of lung cancers (91 / 145) while screening 25.2% (2176 / 8640) of patients.

Association with risk factors and smoking history

The CXR-LC risk score was associated with prevalent risk factors including older age, current smoking, history of COPD, and findings on the radiograph including lung nodules, an enlarged cardiomediastinal silhouette, and edema (Supplemental Figure 11a). CXR-LC estimated risk was associated with pack-year smoking history both above and below the 2022 CMS criteria's 20 pack-year threshold; however, CXR-LC risk was predictive of lung cancer independent of pack-years (Supplemental Figure 11b).

© 2022 Raghu VK et al. *JAMA Network Open*.

eReferences.

[1] Nalichowski R, Keogh D, Chueh HC, Murphy SN. Calculating the benefits of a Research Patient Data Registry. *AMIA Annu Symp Proc.* 2006:1044.

[2] DeLong ER, DeLong DM, Clarke-Pearson DL. Comparing the areas under two or more correlated receiver operating characteristic curves: a nonparametric approach. *Biometrics*. 1988;44(3):837-845.

[3] The Integrated Calibration Index (ICI) and related metrics for quantifying the calibration of logistic regression models. *Stat Med*. 2019;38(21):4051-4065.

[4] Tammemagi MC, Katki HA, Hocking WG, et al. Selection criteria for lung cancer screening. *N Engl J Med*. 2013;368(8):728-736.

[5] Lu MT, Raghu VK, Mayrhofer T, Aerts HJWL, Hoffmann U. Deep learning using chest radiographs to identify high-risk smokers for lung cancer screening computed tomography: development and validation of a prediction model. *Annals of Internal Medicine*. 2020;173(9):704-713.

[6] Irvin J, Rajpurkar P, Ko M, et al. Chexpert: A large chest radiograph dataset with uncertainty labels and expert comparison. 2019.

© 2022 Raghu VK et al. *JAMA Network Open*.

eTable 1. Demographics, Smoking History, Lung Cancer Screening and Outcomes of Patients in the Current Study Cohort, and in the Original Model Development Cohort

	Current Study – External Validation (N = 14,737)	Prostate, Lung, Colorectal, and Ovarian Cancer Screening Trial – Model Development (N = 22,711)
Mean age (SD), y	62.6 (6.8)	62.2 (5.3)
Male sex, n/N (%)	7154 / 14737 (48.5%)	13,660 / 22,711 (60.1%)
Race, n/N(%)		
White	12330 / 14473 (85.2%)	19,797 / 22,711 (87.2%)
Black	1051 / 14473 (7.3%)	1,411 / 22,711 (6.2%)
Asian	204 / 14473 (1.4%)	1,043 (22,711) (4.6%)*
Other	456 / 14473 (3.2%)	
Hispanic Ethnicity, n/N (%)	432/14737 (2.9 %)	460 / 22,711 (2.0%)
Current Smoking, n/N (%)	3433/12807 (26.8%)	4,392 / 22,711 (19.3%)
Mean years since smoking cessation (SD)	19.5 (13.2)	20.4 (11.9)
Mean pack-years (SD)	18.6 (23.5)	35.2 (29.0)
2022 CMS lung cancer screening-Eligible (%)	1151 / 6277 (18.3%)	9852 / 22694 (43.4%)
2015 CMS-Eligible (%)	714 / 7755 (9.2%)	8,456 / 22,711 (37.2%)
6-year lung cancer screening rate (%)	501 / 14737 (3.4%)	N/A
Lung cancer screening rate among 2015-CMS Eligible	84 / 714 (11.8%)	N/A
6-year lung cancer incidence (%)	361/14737 (2.4%)	418 / 22,711 (1.8%)
6-year all-cause mortality, n/N (%)	1284/14737 (8.7%)	1,201 / 22,711 (5.3%)
Lung nodule present on radiograph, n/N (%)	1662/14737 (11.3 %)	2,208/22,711 (9.7%)
Presence of COPD and Emphysema	2039/14737 (13.8%)	869/22,640 (3.8%)

© 2022 Raghu VK et al. *JAMA Network Open*.

eFigure 1. Lung Cancer Rates by 2015 CMS and 2022 CMS Eligibility Criteria

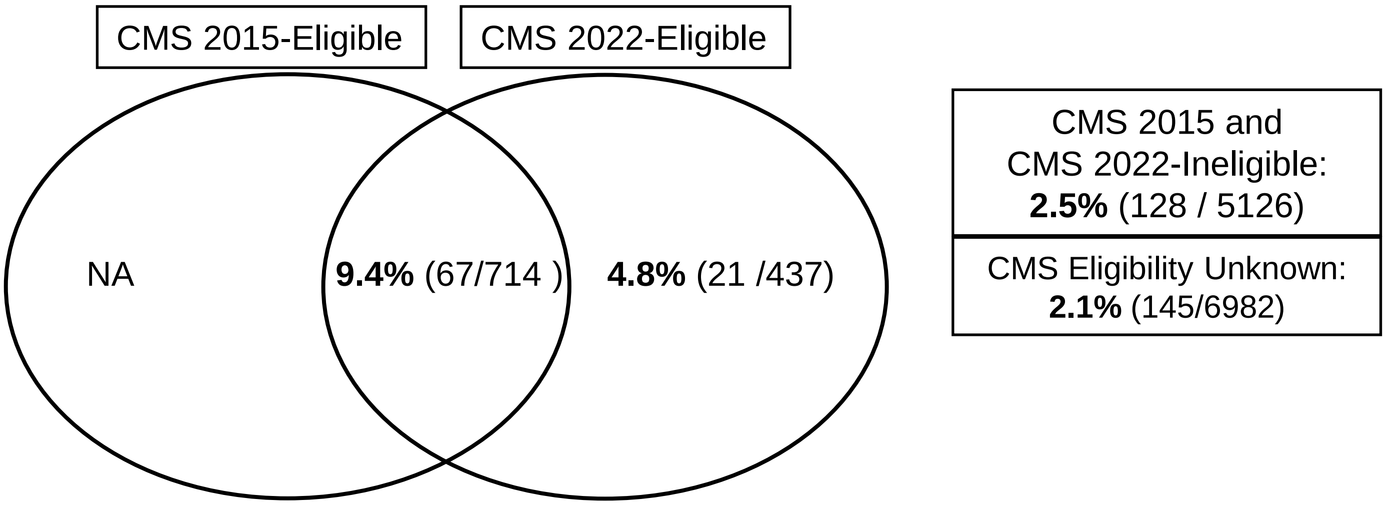

© 2022 Raghu VK et al. *JAMA Network Open*.

eTable 2. Discrimination for 6-year Incident Lung Cancer

	AUC (95% CI) Smoking History Unavailable (N = 8,460)	AUC (95% CI) Smoking History Available (N = 6,277)	AUC (95% CI) Excluding patients with lung nodules and lung cancers within 9 months (N = 5,608)
CXR-LC	0.740 (0.70,0.78)	0.692 (0.66,0.73)	0.704 (0.67,0.74)
2015 CMS Eligibility		0.602 (0.57,0.63)***	0.597 (0.56,0.63)***
2022 CMS Eligibility		0.616 (0.58,0.65)***	0.623 (0.58,0.66)**

*** $p < 0.001$ for difference from CXR-LC
** $p < 0.01$ for difference from CXR-LC
* $p < 0.05$ for difference from CXR-LC

© 2022 Raghu VK et al. *JAMA Network Open*.

eTable 3. Discrimination for 6-year Incident Lung Cancer in Subgroups by Sex and Self-reported Race

	Female Sex		Male Sex		Black race	
	AUC (95% CI) Smoking History Unavailable (N = 4,259)	AUC (95% CI) Smoking History Available (N = 3,324)	AUC (95% CI) Smoking History Unavailable (N = 4,201)	AUC (95% CI) Smoking History Available (N = 2,953)	AUC (95% CI) Smoking History Unavailable (N = 531)	AUC (95% CI) Smoking History Available (N = 522)
CXR-LC	0.751 (0.70,0.81)	0.704 (0.66,0.75)	0.731 (0.67,0.79)	0.679 (0.63,0.73)	0.807 (0.64,0.97)	0.733 (0.51,0.79)
2015 CMS Eligibility		0.596 (0.56,0.64) ***		0.610 (0.56,0.66)*		0.516 (0.44,0.59)**
2022 CMS Eligibility		0.620 (0.58,0.66)**		0.612 (0.56,0.66)*		0.561 (0.45,0.67)*

*** p < 0.001 for difference from CXR-LC
** p < 0.01 for difference from CXR-LC
* p < 0.05 for difference from CXR-LC

© 2022 Raghu VK et al. *JAMA Network Open*.

eFigure 2. Absolute Calibration Plots of CXR-LC Estimated Risk vs Observed Lung Cancer Rate

Dashed line represents perfect calibration.

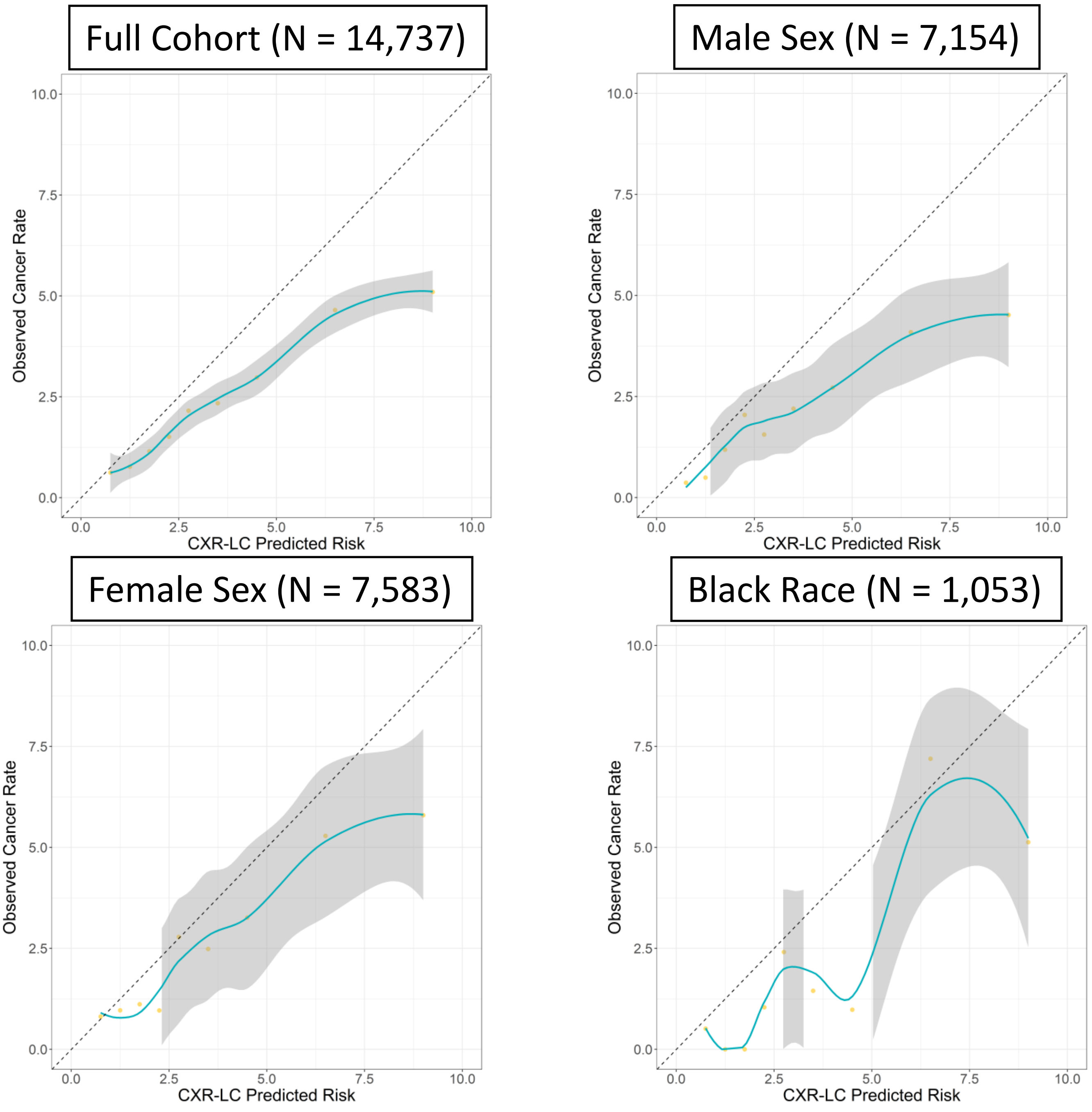

© 2022 Raghu VK et al. *JAMA Network Open*.

eFigure 3. Relative Calibration Plots of CXR-LC Estimated Risk vs. Percentage Calibration Error of Observed Lung Cancers

Dashed line represents perfect calibration.

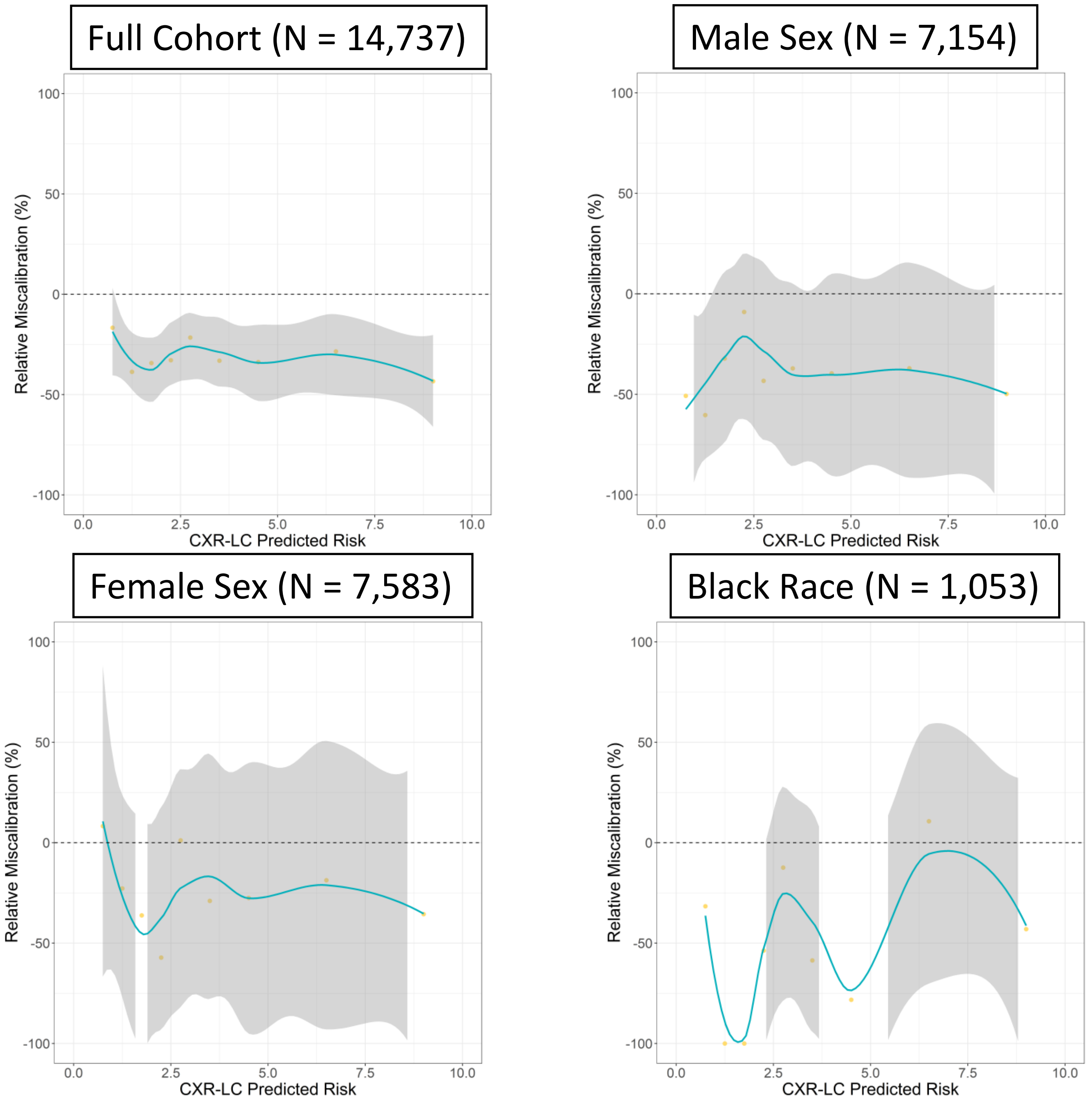

© 2022 Raghu VK et al. *JAMA Network Open*.

eFigure 4. Association Between Ordinal CXR-LC Risk Groups and 6-year Lung Cancer Risk

Cumulative lung cancer incidence by ordinal CXR-LC risk groups (left) and hazard ratios for 6-year lung cancer by CXR-LC risk group (right) calculated using cox proportional hazards regression adjusted for race, sex, history of COPD, and 2022 CMS eligibility

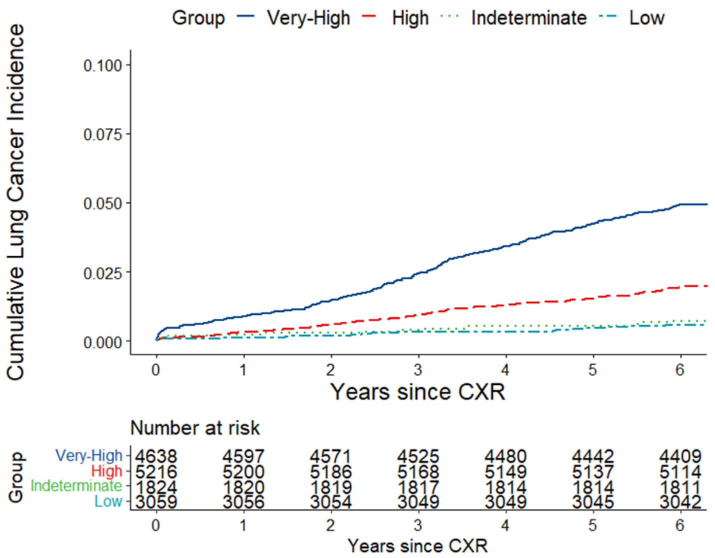

CXRLC-Group	6-Year lung cancers	HR (95% CI)	Adj HR (95% CI)	p-value for Adj HR
Low	8 / 967 (0.8%)	Ref	Ref	NA
Indeterminate	4 / 633 (0.6%)	1.28 (0.6,2.6)	0.73 (0.2,2.4)	0.603
High	66 / 2215 (3.0 %)	3.54 (2.1,5.9)	3.14 (1.5,6.6)	0.002
Very-High	138 / 2462 (5.6%)	9.09 (5.6,14.9)	4.87 (2.4,10.1)	<0.001

© 2022 Raghu VK et al. *JAMA Network Open*.

eTable 4. Six-year Lung Cancer Rates by CXR-LC and 2022 CMS Eligibility Criteria Across Model Development And Validation Cohorts

		CXR-LC Eligible		CXR-LC Ineligible	
**Cohort**	**Description**	**2022 CMS Eligible**	**2022 CMS Ineligible**	**2022 CMS Eligible**	**2022 CMS Ineligible**
**PLCO Internal Testing***	**US Adults 55-74 years of age enrolled at 10 US sites from 1993-2001. Only those with history of smoking included.**	3.1% (43 / 1376)	2.9% (22 / 750)	1.6% (17 / 1,092)	0.4% (9/2397)
**NLST External Validation***	**US Adults 55-74 years of age enrolled at 33 US sites from 2002-2004, ≥30 pack-year smoking history only**	5.1% (172 / 3343)	NA	1.6% (34 / 2150)	NA
**MGB External Validation**	**Adults 50-80 years of age with a chest radiograph taken at a Mass General Brigham site in 2013-2014**	8.5% (83 / 974)	3.3% (121 / 3703)	2.8% (5 / 177)	0.5% (7 / 1423)

*previously reported in Lu MT, Raghu VK, Mayrhofer T, Aerts HJWL and Hoffmann U. Deep learning using chest radiographs to identify high-risk smokers for lung cancer screening CT: Development and validation of a prediction model. Annals of Internal Medicine. 2020 Sep 1;173(9):704-13.

© 2022 Raghu VK et al. *JAMA Network Open*.

eFigure 5. Lung Cancer Rates by CXR-LC and 2022 CMS Eligibility Criteria, Removing Positive Radiograph Screens and Those With Confirmed Lung Cancer Within 9 Months

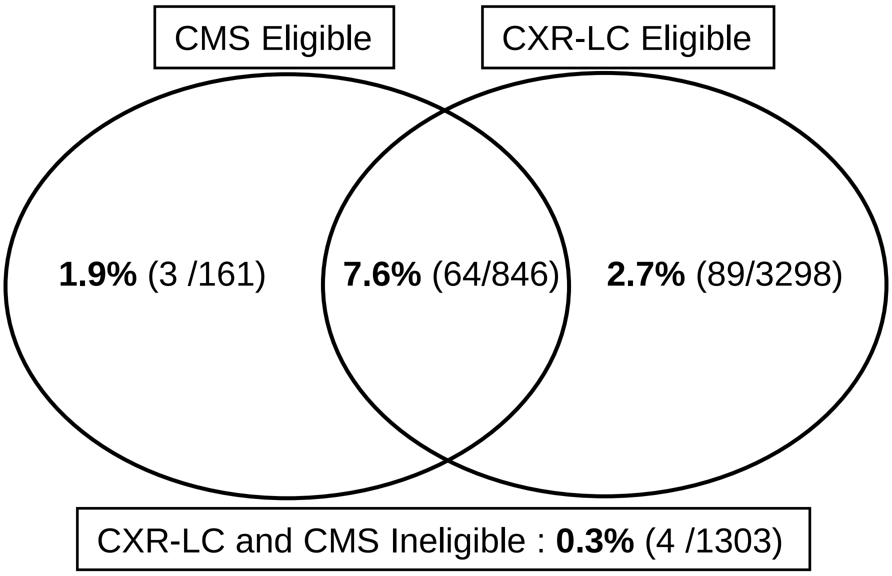

	CXR-LC Eligible	CXR-LC Ineligible
CMS Eligibility Unknown	**2.1%** (94/4470)	**0.4%** (11 /2970)

© 2022 Raghu VK et al. *JAMA Network Open*.

eFigure 6. Lung Cancer Rates by CXR-LC and 2022 CMS Eligibility Criteria in Black Patients

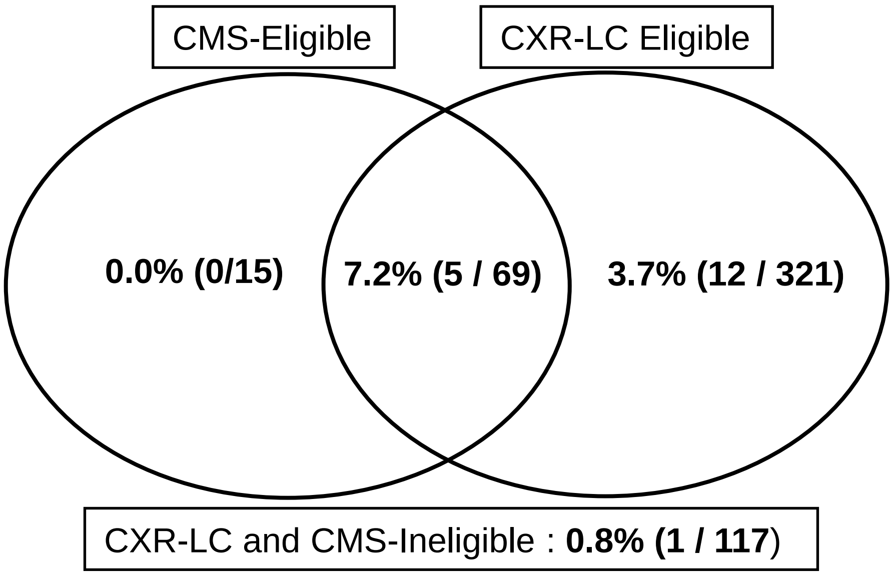

	CXR-LC Eligible	CXR-LC Ineligible
CMS Eligibility Unknown	1.0% (3 / 305)	0.0% (0 / 226)

© 2022 Raghu VK et al. *JAMA Network Open*.

eFigure 7. Cumulative Lung Cancer Incidence by CXR-LC and CMS Eligibility Criteria

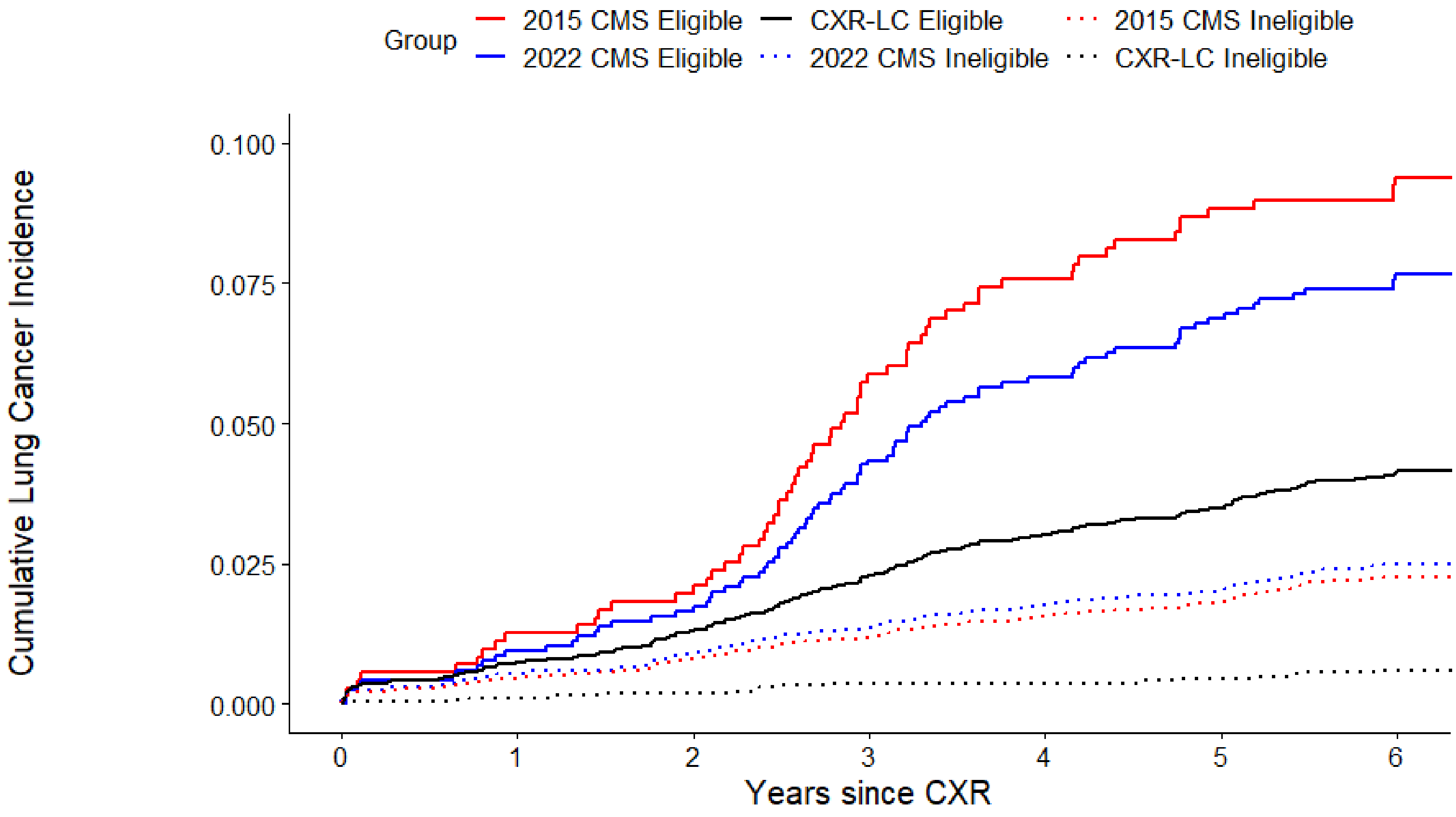

Number at risk

Group	0	1	2	3	4	5	6
2015 CMS Eligible	714	705	700	672	660	651	647
2022 CMS Eligible	1151	1140	1132	1101	1084	1072	1063
CXR-LC Eligible	5089	5051	5023	4973	4936	4911	4878
2022 CMS Ineligible	5126	5098	5079	5056	5036	5023	4998
2015 CMS Ineligible	7041	7009	6984	6957	6932	6914	6881
CXR-LC Ineligible	2666	2663	2661	2656	2656	2654	2650

Years since CXR

© 2022 Raghu VK et al. *JAMA Network Open*.

eTable 5. Test Statistics for CXR-LC and 2015 and 2022 CMS Eligibility Criteria for 6-year Incident Lung Cancer in Black Patients With Smoking History

	**2015 CMS Eligibility**	**2022 CMS Eligibility**	**CXR-LC Eligibility**
Smoking History Available (n=522)			
Sensitivity (95% CI), %	11.1 (1.4,34.7)	27.8 (9.7,53.5)	94.4 (72.7,99.9)
Specificity (95% CI), %	92.1 (89.3,94.3)	84.3 (80.9, 87.4)	26.0 (22.2,30.1)
PPV (95% CI), %	4.8 (0.1,16.2)	6.0 (2.0, 13.3)	4.4 (2.6,6.9)
NPV (95% CI), %	96.7 (94.6,98.1)	97.0 (95.0,98.4)	99.2 (95.9,100.0)
Participants eligible for screening, n (%)	42 / 522 (8.0%)	84 / 522 (16.1%)	390 / 522 (74.7%)
Lung cancers included, n (%)	2 / 18 (11.1%)	5 / 18 (27.8%)	17 / 18 (94.4%)
Smoking History Unavailable (n=531)			
Sensitivity (95% CI), %	N/A		100.0 (29.2,100.0)
Specificity (95% CI), %			42.8 (38.5,47.1)
PPV (95% CI), %			1.0 (0.2,2.8)
NPV (95% CI), %			100.0 (98.4,100.0)
Participants eligible for screening, n (%)			305 / 531 (57.4%)
Lung cancers included, n (%)			3 / 3 (100.0%)

© 2022 Raghu VK et al. *JAMA Network Open*.

eTable 6. Test Statistics for 6-year Lung Cancer Rate by 2015 and 2022 CMS Eligibility and CXR-LC Using a Risk Threshold to Match the Specificity of 2022 CMS Criteria

	**2015 CMS Eligibility**	**2022 CMS Eligibility**	**CXR-LC Matched Specificity**
Smoking History Available (n=6277)			
Sensitivity (95% CI), %	31.0 (24.9-37.6)	40.7 (34.1-47.6)	40.3 (33.7-47.1)
Specificity (95% CI), %	89.3 (88.5-90.1)	82.5 (81.5-83.4)	82.5 (81.5-83.4)
PPV (95% CI), %	9.4 (7.3-11.8)	7.6 (6.2-9.3)	7.6 (6.1-9.2)
NPV (95% CI), %	97.3 (96.9-97.7)	97.5 (97.0-97.9)	97.5 (97.0-97.9)
Participants eligible for screening, n (%)	714 / 6277 (11.4)	1151 / 6277 (18.3)	1151 / 6277 (18.3)
Lung cancers included, n (%)	67 / 216 (31)	88 / 216 (40.7)	87 / 216 (40.3)
Smoking History Unavailable (n=8460)			
Sensitivity (95% CI), %	N/A		27.6 (20.5-35.6)
Specificity (95% CI), %			91.3 (90.6 – 91.9)
PPV (95% CI), %			5.2 (3.8 – 7.0)
NPV (95% CI), %			98.6 (98.4 – 98.9)
Participants eligible for screening, n (%)			767 / 8640 (8.9)
Lung cancers included, n (%)			40 / 145 (27.6)

© 2022 Raghu VK et al. *JAMA Network Open*.

eFigure 8. Lung Cancer Rates by CXR-LC Very-High and 2022 CMS Eligibility Criteria

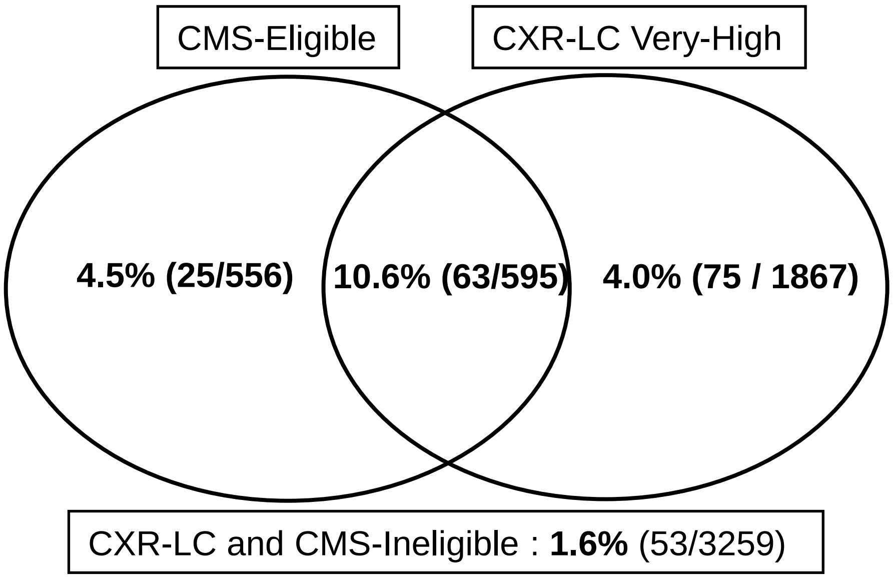

	CXR-LC Very High	CXR-LC High or Ineligible
CMS Eligibility Unknown	**4.2%** (91 / 2176)	**0.9%** (54 / 6284)

© 2022 Raghu VK et al. *JAMA Network Open*.

eFigure 9. Lung Cancer Rates by CXR-LC Very-High and 2022 CMS Eligibility Criteria Removing Positive Radiograph Screens and Those With Confirmed Lung Cancer Within 9 Months

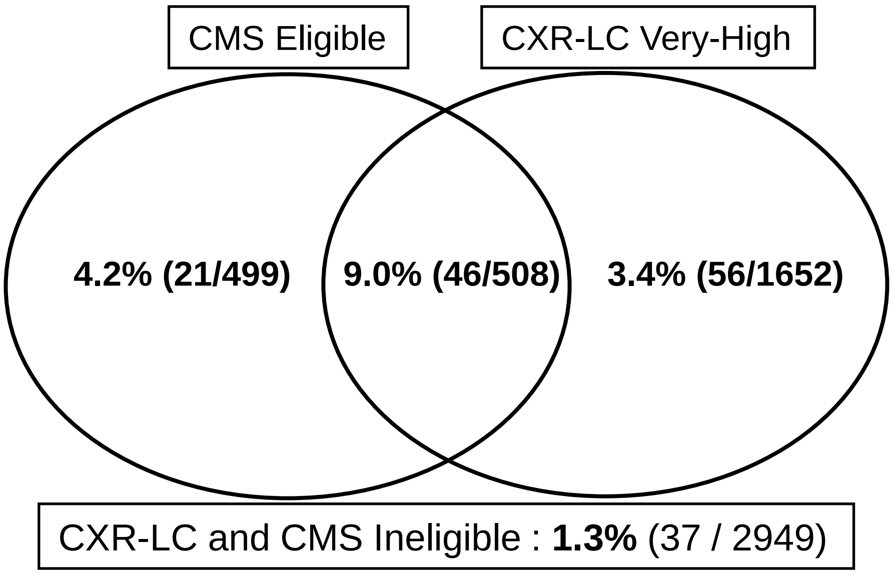

	CXR-LC Very-High	CXR-LC Ineligible/High
CMS Eligibility Unknown	3.5% (64/1855)	0.7% (41/5585)

© 2022 Raghu VK et al. *JAMA Network Open*.

eFigure 10. Lung Cancer Rates by CXR-LC Very-High and 2022 CMS Eligibility Criteria in Black Patients

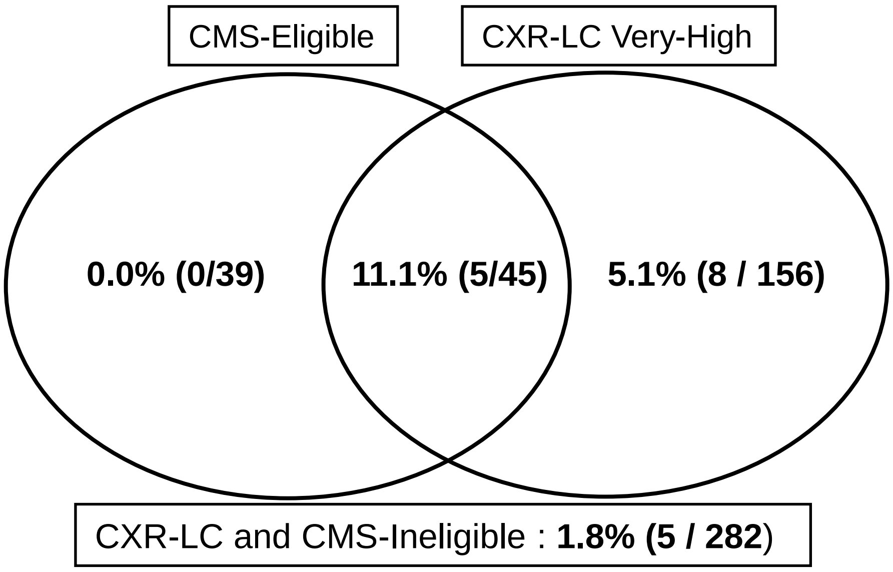

	CXR-LC Very-High	CXR-LC High or Ineligible
CMS Eligibility Unknown	1.7% (2/118)	0.2% (1 / 413)

© 2022 Raghu VK et al. *JAMA Network Open*.

eFigure 11. Spearman Correlation Between the CXR-LC Probability Output and Prevalent Risk Factors and Pack-year Smoking History vs. CXR-LC Risk Probability

This plot shows only correlations between the raw CXR-LC probability score with prevalent risk factors and imaging findings. The only inputs to the CXR-LC model are age, sex, current smoking, and a CXR image.

A. CXR-LC probability and prevalent risk factors

Risk Factor	Positive Cases (%)	Correlation	p-value
Current Smoking	3169 (21.5%)	0.44	<0.001
Age	NA	0.43	<0.001
Prevalent COPD	2039 (13.8%)	0.3	<0.001
Support Devices	2344 (15.9%)	0.1	<0.001
Pack Years	NA	0.09	<0.001
Male Sex	7154 (48.5%)	0.08	<0.001
Edema	659 (4.5%)	0.07	<0.001
Atelectasis	1734 (11.8%)	0.07	<0.001
Enlarged Cardiomediastinum	8281 (56.2%)	0.07	<0.001
Fracture	808 (5.5%)	0.06	<0.001
Pleural Effusion	884 (6%)	0.06	<0.001
Cardiomegaly	1207 (8.2%)	0.06	<0.001
Lung Nodule	1662 (11.3%)	0.05	<0.001
Pneumonia	1480 (10%)	0.04	<0.001
Consolidation	379 (2.6%)	0.03	0.001
Pneumothorax	327 (2.2%)	-0.01	0.2
Years since quit smoking	NA	-0.17	<0.001

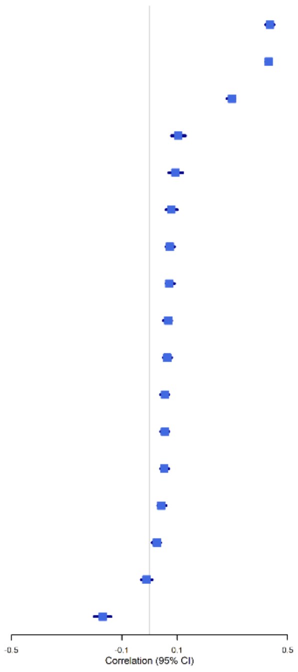

B. Pack-year vs CXR-LC risk probabaility

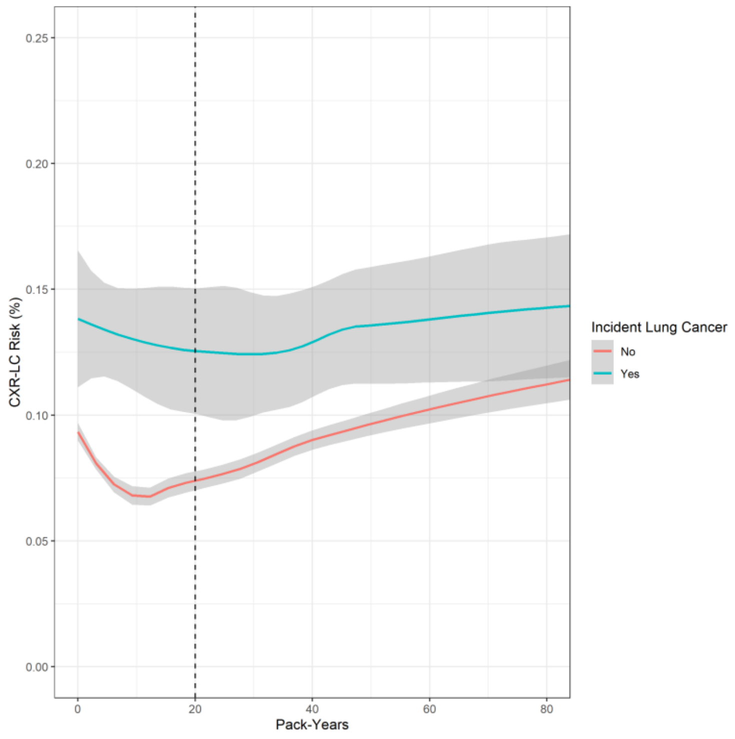

© 2022 Raghu VK et al. *JAMA Network Open*.
